# Hazard Recognition Patterns Demonstrated by Construction Workers

**DOI:** 10.3390/ijerph17217788

**Published:** 2020-10-24

**Authors:** S M Jamil Uddin, Alex Albert, Abdullah Alsharef, Bhavana Pandit, Yashwardhan Patil, Chukwuma Nnaji

**Affiliations:** 1 Department of Civil, Construction, and Environmental Engineering, North Carolina State University, 2501 Stinson Dr., Raleigh, NC 27607, USA; alex_albert@ncsu.edu (A.A.); afalshar@ncsu.edu (A.A.); bkpandit@ncsu.edu (B.P.); yspatil@ncsu.edu (Y.P.); 2 Department of Civil, Construction, and Environmental Engineering, The University of Alabama, 3023 HM Comer, Tuscaloosa, AL 35487, USA; cnnaji@eng.ua.edu

**Keywords:** construction safety, hazard recognition, occupational safety, worker safety, hazard recognition pattern, construction hazards, safety risks

## Abstract

Construction workers fail to recognize a large number of safety hazards. These unrecognized safety hazards can lead to unintended hazard exposure and tragic safety incidents. Unfortunately, traditional hazard recognition interventions (e.g., job hazard analyses and safety training) have been unable to tackle the industry-wide problem of poor hazard recognition levels. In fact, emerging evidence has demonstrated that traditional hazard recognition interventions have been designed without a proper understanding of the challenges workers experience during hazard recognition efforts. Interventions and industry-wide efforts designed based on a more thorough understanding of these challenges can yield substantial benefits—including superior hazard recognition levels and lower injury rates. Towards achieving this goal, the current investigation focused on identifying hazard categories that workers are more proficient in recognizing and others that they are less proficient in recognizing (i.e., hazard recognition patterns). For the purpose of the current study, hazards were classified on the basis of the energy source per Haddon’s energy release theory (e.g., gravity, motion, electrical, chemical, etc.). As part of the study, 287 workers representing 57 construction workplaces in the United States were engaged in a hazard recognition activity. Apart from confirming previous research findings that workers fail to recognize a disproportionate number of safety hazards, the results demonstrate that the workers are more proficient in recognizing certain hazard types. More specifically, the workers on average recognized roughly 47% of the safety hazards in the gravity, electrical, motion, and temperature hazard categories while only recognizing less than 10% of the hazards in the pressure, chemical, and radiation hazard categories. These findings can inform the development of more robust interventions and industry-wide initiatives to tackle the issue of poor hazard recognition levels in the construction industry.

## 1. Introduction and Study Motivation

Poor safety performance is a universal problem in construction workplaces [1,2,3,4,5,6]. For example, global estimates suggest that more than 60,000 fatalities and many more non-fatal safety incidents are experienced in construction workplaces every year [7,8]. In the United States, over 1000 fatalities and 200,000 non-fatal incidents have been reported in recent years [1]. These injuries cause much physical, emotional, and financial distress for workers and their families [9]. The injuries also result in unnecessary costs that exceed millions of dollars, which in many cases threaten the survival of construction businesses [10,11].

One explanation for such poor performance highlighted in recent research is the prevalence of poor hazard recognition levels in construction workplaces [12,13,14]. When workers fail to recognize safety hazards, the likelihood of unintended hazard exposure and tragic safety incidents increases substantially [12,14]. On the other hand, if workers recognize safety hazards, they are more likely to effectively manage safety hazards and reduce injury likelihood. Unfortunately, evidence from the global construction industry has unanimously demonstrated that poor hazard recognition is a universal problem across nations and workplaces [12,13,14,15,16,17,18,19,20].

Since hazard recognition is fundamental to effective safety management, most workplaces adopt a number of interventions to facilitate proper hazard recognition [19,21,22,23]. Nonetheless, recent research has demonstrated that a significant number of hazards continue to remain unrecognized even when these interventions are adopted [24]. These findings have unveiled important weaknesses within existing and widely adopted hazard recognition interventions [22]. In fact, there is evidence to suggest that much of the hazard recognition interventions have been developed without a sufficient understanding of the challenges associated with hazard recognition in dynamic and rapidly changing environments [24]. A thorough understanding of these challenges can be strategically leveraged to develop more robust and failure-proof hazard recognition interventions.

Towards achieving this goal, the current investigation focused on unveiling hazard recognition patterns that workers demonstrate during hazard recognition efforts. More specifically, the research focused on understating hazard types that workers are more proficient in recognizing and others that they are less proficient in recognizing. The investigation was undertaken to better understand hazard categories that workers are more likely to struggle with recognizing in complex work environments.

## 2. Background

### 2.1. Significance of Construction Hazard Recognition

Preventing hazard exposure is fundamental to construction injury and illness prevention [25,26]. To prevent hazard exposure, safety hazards must first be recognized before they can be avoided or managed [20]. When safety hazards are not recognized, they are also likely to remain unmanaged—which can increase the likelihood of workplace incidents and injuries [12,14]. Therefore, hazard recognition is largely regarded as one of the most fundamental steps and a pre-requisite to injury prevention.

Unfortunately, a large body of literature has demonstrated that workers fail to recognize a substantial number of safety hazards. For example, an investigation from the United States revealed that workers fail to recognize over 40% of safety hazards [12]. Another study from Australia found that workers may fail to recognize up to 57% of safety hazards [13]. Yet, another study from the United Kingdom found that individuals may fail to recognize up to 33% of relevant safety hazards [14]. Similar and more recent replications have also reinforced these previous findings in the context of nations including Asia, the Middle East, and other geographical regions [12,13,14,15,16,17,18,20,27]. 

Previous research has also linked poor hazard recognition with higher injury rates [28]. In fact, the Occupational Safety and Health Administration (OSHA) has labeled unrecognized hazards as a “root cause” for injuries, illness, and safety incidents [26]. Therefore, efforts to enhance hazard recognition are essential to addressing the safety challenges experienced in the construction industry. The following section describes some of the interventions that are widely adopted across the construction industry in an effort to improve hazard recognition levels.

### 2.2. Construction Hazard Recognition Interventions

Due to the importance of hazard recognition in injury prevention, several interventions are adopted in construction workplaces to promote hazard recognition levels. Examples of these interventions include safety training efforts, adoption of job hazard analysis protocols, and the use of safety checklists [19,21,22,29]. 

For example, the industry has invested millions of dollars in offering safety training to enhance hazard recognition and management levels [19,29]. Unfortunately, desirable levels of return have not been achieved from these investments for a variety of reasons [19,30]. One major reason discussed in the broader literature is that traditional safety training programs, although widely adopted, are not designed with a sufficient understanding of why workers fail to recognize safety hazards [24]. Further, traditional safety training interventions lack the integration of robust and personalized training protocols [31]. Other reasons include the persuasiveness of training programs that do not sufficiently engage workers [19,32], training programs that adopt pedagogical instructional methods as opposed to andragogical approaches—which are more suitable for adult learners [32,33,34]—and the lack of measures to foster training transfer where workers adopt learned concepts after they return to the field [19,35]. 

While job hazard analysis (JHA) and safety checklists [21,22,36] offer their own advantages, these interventions also have important and embedded weaknesses that result in unrecognized safety hazards [37]. For example, the job hazard analyses approach assumes that workers are able to accurately predict conditions that will emerge as work progresses and that workers have the innate ability to report relevant safety hazards. However, recent investigations have demonstrated that workers often are unable to accurately predict future conditions, particularly in dynamic and rapidly changing environments, and workers do not possess the innate ability to recognize all relevant safety hazards that can cause harm [38,39,40]. Past research has also highlighted that JHAs are unable to capture hazards created by other crews, since JHAs are largely prepared at the crew level—without any collaboration with members of other crews that may work in the same area [22].

Safety checklists, on the other hand, offer a template that includes a list of pre-defined safety hazards to guide the hazard recognition process [41]. Unfortunately, such pre-defined lists are often not comprehensive of all possible safety hazards in dynamic work environments [42]. Moreover, past research has argued that the use of safety checklists can lead to tunnel vision—where the workers only look for safety hazards that are included as part of the safety checklist templates [43]. Others have also suggested that the use of safety checklists can give a false sense of security to individuals when the workplace complies with the requirements as set by the checklist—even though there may be other unaddressed safety issues that are outside the scope or coverage of the checklist [40,44].

Due to such weaknesses with traditional interventions, workers often fail to recognize important safety hazards that can cause harm and injury. To counter these issues, hazard recognition interventions that are cognizant of the challenges that workers experience during hazard recognition efforts are necessary. Such new interventions can promote hazard recognition and reduce safety incidents.

## 3. Research Objectives and Contributions

Although the problem of poor hazard recognition levels and the weaknesses of existing interventions are now widely acknowledged in the broader literature as discussed in the previous sections, there has not been much work that has focused on addressing these issues. There is a need to better understand why workers fail to recognize safety hazards, what types of hazards they generally fail to recognize, and what improvements can be made to existing interventions to address the prevalence of poor hazard recognition levels. 

To advance relevant research, the current investigation focused on understanding the hazard recognition patterns that workers demonstrate when tasked with participating in hazard recognition efforts. More specifically, the investigation focused on understanding the hazard types that workers are more proficient in recognizing and others that they are less proficient in recognizing. Such an investigation can inform the development of more robust hazard recognition interventions that target particular hazards that are more likely to remain unrecognized. Moreover, such an investigation can offer important insights into problem areas and challenges that workers experience as they participate in hazard recognition efforts—for which robust solutions will need to be developed. Unlike previous efforts that examined the effect of factors such as safety climate [45], worker experience [17], and other factors [16,43] on hazard recognition levels and related safety outcomes [45], the current investigation examined if workers demonstrate any disparities in recognizing hazards of different types or categories.

For the purpose of this investigation, hazard types or hazard categories were operationalized in accordance with the energy release theory pioneered by Haddon (1973) [46]—which was later customized for the needs of the construction industry—as reported by Fleming [40] and Albert et al. [12]. As per this approach, all workplace safety incidents and injuries can be traced back to the improper release and exposure to specific hazardous energy sources. Accordingly, hazards can be classified on the basis of these energy sources as shown in Table 1. Table 1 also includes a few construction-specific hazard examples that correspond to each of the energy sources for illustrative purposes. Using these hazard types as the underlying framework, the current investigation focused on examining if there are any differences in the proficiency with which workers recognize safety hazards across the hazard categories presented in Table 1. 

As can be seen, the scope of the hazards examined in the current effort is limited to physical hazards that can be observed during a site examination. The study does not include non-physical hazards such as psychological hazards that may include stress and workplace conflicts. The scope of the study also does not directly examine upstream factors such as management role, workplace pressure, team support, and other factors that can impact workplace safety.

## 4. Material and Methods

To accomplish the research goals, it was necessary first to develop a standardized and reliable hazard recognition activity that can be administered to construction workers. This was accomplished by leveraging 16 construction case images that were captured during visits of real construction workplaces in the United States as part of a previous research investigation [12]. The case images captured a wide variety of construction operations that included gas welding, crane rigging and lifting, cutting, welding, drilling, and others. 

After the case images were captured, as part of the previous effort, the images were examined collaboratively by a group of 17 construction industry safety professionals that possessed a collective experience of over 300 years. The purpose of the examination was to enlist all safety hazards that were represented in the case images. The examination was guided using the energy sources that are listed in Table 1. Accordingly, the safety hazards along with the relevant energy sources were recorded as part of the previous investigation [12].

The examination effort yielded a total of 120 safety hazards across the 16 case images with each of the images including at least five safety hazards. An example case image that includes the pre-identified safety hazards along with the corresponding energy source or hazard categories is reproduced from Pandit et al. [45] for illustration purposes.

While the case images included hazards that corresponded to each of the 10 hazard categories presented in Table 1, few hazard categories were under-represented in the case images. These included biological hazards that included only 2 relevant hazards, mechanical hazards that included only 2 hazards, and sound hazard that included only 1 hazard across the case images. Since the objective of the current investigation was to make reliable inferences with regards to the categories of safety hazards rather than a few or particular safety hazards of interest, these under-represented hazard categories were excluded from the current investigation. Therefore, the study examined 7 hazard categories that included at least 5 independent safety hazards (i.e., Gravity—46; Motion 27; Electrical—9; Pressure—5; Temperature—10; Chemical—13; and Radiation—5), encompassing a total of 115 safety hazards after the exclusion of the five hazards that represented the under-represented hazard categories.

Having finalized the case images and the scope of the investigation, a convenience sample of 287 workers representing 57 workplaces in the United States was recruited to participate in a hazard recognition activity. The workers were involved in a variety of trades including plumbing, carpentry, electrical works, and others and represented projects focusing on a mix of infrastructure, industrial, commercial, and residential development projects. The experience of the recruited workers in the construction industry ranged between 1 and 40 years, with an average of 13 years.

From the set of 16 case images discussed above, each of the recruited workers was presented with a random set of two case images. The workers were then tasked with examining the case images and reporting all relevant safety hazards. Across the case images examined by each of the 287 participating workers, there were a total of 4106 safety hazards that the workers could potentially report that were within the scope of the examined hazard categories (i.e., after the under-represented hazard categories were excluded). Based on the gathered data, the hazard categories that the workers were proficient in recognizing were determined using the approach outlined in the following section.

## 5. Data Handling, Analysis, and Results

**Figure 1:**
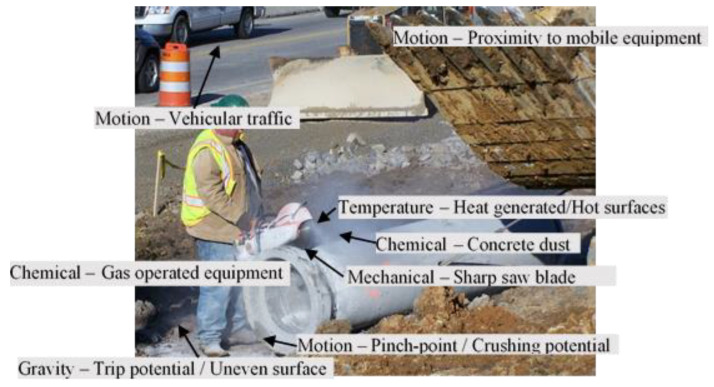
Example of a case image with pre-identified safety hazards.

Once the data were gathered, the proficiency with which the workers recognized each of the safety hazards (*HR_p_*) was first calculated using Equation (1)—which simply represented the proportion of workers that recognized each of the 115 safety hazards. For example, as part of the study, the case image presented as Figure 1 was examined by 34 workers following the random assignment of the case images. Of the 34 workers who could potentially recognize the motion-related safety hazard of being in the proximity of a mobile equipment (see Figure 1), 27 workers demonstrated proficiency by recognizing and reporting the hazard. In other words, the motion-related hazard involving the proximity to a mobile equipment was recognized by 79.41% (i.e., 27 of a total of 34) of the workers that examined the case image. Accordingly, as per Equation (1), the proportion of workers that recognized each of the safety hazards could potentially range from as low as 0%, where none of the workers presented with a particular hazard recognized the specific hazard, to 100%, where all the workers presented with a particular hazard recognized the hazard.

(1) $$HR_{p}=\frac{No.\;of\;workers\;\mathrm{that}\;\mathrm{recogized}\;a\;\mathrm{particular}\;\mathrm{hazard}}{No\;of\;workers\;\mathrm{that}\;\mathrm{examml:mined}\;\mathrm{the}\;\mathrm{case}\;\mathrm{image}\;\mathrm{that}\;\mathrm{included}\;\mathrm{the}\;\mathrm{particular}\;\mathrm{hazard}}$$

Having computed the proportion of workers that recognized each of the 115 safety hazards that were within the scope of the study, the hazards were separated on the basis of the pre-identified hazard categories (i.e., Gravity—46; Motion 27; Electrical—9; Pressure—5; Temperature—10; Chemical—13; and Radiation—5). This was followed by the calculation of the descriptive statistics that provided the demonstrated proficiency levels for each of the hazard categories as presented in Table 2. As can be seen, descriptively, the workers demonstrated relatively higher levels of proficiency in recognizing hazards in the gravity and electrical categories. More specifically, on average, the workers recognized more than 60% of the gravity hazards and 44% of the electrical hazards. This was followed by temperature and motion hazards, which were both associated with a proficiency level that exceeded 42%. There was relatively poor proficiency in recognizing pressure, chemical, and radiation hazards, where workers demonstrated a proficiency that was slightly above 15%, 8%, and 5%, respectively.

It is important to note that the study design and the data analysis approach were designed to compare the performance of the same group of study participants across the hazard categories. In other words, although the relative performance was assessed across the hazard categories of interest (e.g., gravity, motion, electrical, etc.), the participants themselves remained the same across the hazard categories. Therefore, the study design and analysis plan efficiently controlled for any individual differences among the study participants—such as age, experience, or previous training. Accordingly, given the nature of the study design and the analysis approach, it was not necessary to statistically control for individual differences (e.g., experience) among the study participants to make valid conclusions. 

While the descriptive statistics demonstrated differences in proficiency levels, as discussed above, the next step was to assess if the observed differences were statistically significant or could be merely attributable to random variability in the data. To make this assessment, the one-way analysis of variance (ANOVA) approach for making statistical inferences was adopted. 

Prior to selecting the most appropriate ANOVA test, the normality of the data was assessed using the Shapiro–Wilk test [47]. The findings offered evidence to suggest that the data in several of the hazard categories were not normally distributed. Next, the homogeneity of the variance across the hazard categories was tested using Levene’s test. Levene’s test results demonstrated that the variance across the hazard categories was unequal.

Given that the data were not normally distributed, had an unequal number of data points under each of the hazard categories, and did not exhibit homogeneity of variance, Welch’s ANOVA was adopted instead of Fisher’s standard ANOVA and the Kruskal–Wallis test [48,49]. The results of the analysis are also presented in Table 2.

While the results presented in Table 2 from Welch’s ANOVA provided evidence that the workers were more proficient in recognizing hazards of certain categories [F(6,24.49) = 28.518, *p* < 0.05), the findings do not reveal the specific hazard categories that workers were statistically more proficient in recognizing. Therefore, a post hoc analysis that makes pairwise comparison of the hazard categories was conducted. Given that the assumption of the homogeneity of variance was violated, as discussed earlier, the Games–Howell post hoc analysis was adopted. Moreover, the bootstrapping approach with 1000 resamples was adopted given that the resampling approach is remarkably robust against data with irregular distributions and the violation of normality [50,51].

The results of the post hoc pairwise comparisons are presented in Table 3. As can be seen, given that the pairwise comparisons involved seven hazard categories, there are a total of 21 unique pairwise comparisons. The mean difference that is included in the table represents the difference in the mean proficiency levels observed when comparing each of the pairwise hazard categories. For example, the mean difference for the Gravity–Motion pairwise comparison is equal to 17.51—which is the difference in the proficiency for the gravity and motion hazard categories (i.e., 60.13%–42.62%) as presented in Table 2.

The bootstrap-based confidence intervals included in Table 3 provide information of the pairwise comparisons that were significantly different. Cases where the confidence interval does not include zero as a possible value for the differences in the pairwise means suggest that the difference is significant. These results suggest that there was a significant difference in the proficiency levels when comparing hazards in the gravity hazard category with hazards in the pressure, chemical, and radiation hazard categories. However, a significant difference was not found when comparing proficiency levels between gravity, electrical, motion, and temperature hazard categories. 

In the same manner, significant differences in proficiency were found between hazards in the motion, electrical, and temperature hazard categories when compared with the hazards in the pressure, chemical, and radiation hazard categories. However, no significant differences in proficiency levels were found when comparisons were performed between motion, electrical, and temperature hazard categories or when comparing pressure, chemical, or radiation hazard categories.

To better visualize the results and findings, Table 4 was created which summarizes the results from Table 3. In the table, the hazard categories that were not significantly different have been grouped under either group A or B. As can be seen in Table 4, although workers recognized a higher proportion of gravity hazards on the basis of the descriptive statistics that are presented in Table 2, the proficiency with which the workers recognized hazards in the gravity, electrical, motion, and temperature categories was statistically equivalent. Likewise, the proficiency level in recognizing hazards in the pressure, chemical, and radiation categories was also statistically equivalent. However, the workers demonstrated higher proficiency levels when examining gravity, electrical, motion, and temperature hazards that are shown to be part of group A when compared to the pressure, chemical, and radiation hazards that are represented under group B.

## 6. Discussion and Study Implications

The findings of the presented study provide useful insights that can be leveraged to improve safety performance in the construction industry. First, the study provided evidence to suggest that workers are less proficient in recognizing certain hazard categories compared to others. More specifically, the study demonstrated that workers are particularly less proficient in recognizing hazards in the pressure, chemical, and radiation hazard categories. Therefore, to ensure that workers are sufficiently protected and to ensure safety excellence, the findings suggest that increased attention may need to be devoted to these hazards that workers are more likely to not recognize. Such efforts are particularly crucial given that the current investigation demonstrates that workers were only able to recognize less than 10% of the hazards that fall under these hazard categories (i.e., average proficiency across pressure, chemical, and radiation hazards). The focus on these hazards is also important given that incidents from these under-recognized hazard categories have been linked with significant levels of risk, injury, and illnesses. For example, pressure-related hazards such as failure of high-pressure pipelines and cave-ins in the context of excavations and trenches have resulted in a disproportionate number of safety incidents [52]. Likewise, the exposure to chemical hazards such as welding fumes, silica, asbestos, and other carcinogens has been highlighted as posing a significant safety risk to construction workers in the broader literature [53,54,55]. In the same manner, radiation-related hazards such as the ultraviolet radiations generated from hot work such as welding and cutting operations, and activities involving working alongside radioactive elements can impose a significant risk to construction workers [56]. To enhance hazard recognition in the context of these under-recognized safety hazard categories, industry leaders, agencies, and employers may focus on emphasizing the risk associated with these hazard types as part of their training efforts. The hazard sources may also be highlighted as part of the job hazard analyses templates that workers use as part of their planning operations to alert workers to these hazard categories that are less proficient in being recognized by workers.

Second, the study demonstrates that workers are more proficient in recognizing certain hazard categories—that include gravity, electrical, motion, and temperature hazards. While the study does not provide any insight into why workers are particularly more proficient in recognizing hazards in these hazard categories, these patterns may be partly attributable to a number of industry initiatives. One of them may be the amount of industry-wide safety initiatives that seek to tackle the hazards that largely fall in these categories [57,58]. An example of such efforts is the nation-wide campaign—called the National Safety Stand-Down to Prevent Falls—organized each year through a collaborative effort by the National Institute for Occupational Safety and Health (NIOSH), the Occupational Safety and Health Administration (OSHA), and the Center for Construction Research and Training (CPWR) [59]. This effort seeks to tackle falls-related hazards which is the leading cause of fatalities in the construction industry—which also happens to be one of the major sources of hazards that fall under the gravity hazard category as presented in the current study. Examples of gravity-related hazards that have been emphasized as part of this program include falls from roofs, falls from ladders, falls from temporary structures such as scaffolds and staging, and falls from aerial lifts, man lifts, and other heavy equipment [60].

Another major industry initiative that targets a large number of hazards that fall under the hazard categories that workers were more proficient in recognizing as demonstrated in the current study is the Construction Focus Four—or more popularly referred to as the Construction Fatal Four training program [61]. This program, pioneered and disseminated across the industry by the Occupational Safety and Health Administration (OSHA), seeks to promote hazard recognition and management in the context of the most common causes of fatal incidents in the construction industry—which include falls, caught-in or between, struck-by, and electrocution incidents. Examples of hazards highlighted as part of this program include falls from ladders and unprotected edges that fall under the gravity hazard category; electrocution from power tools and overhead powerlines that fall under the electrical hazard category; and struck-by incidents involving heavy equipment and swinging loads that are within the scope of motion-related hazards. However, it is important to note the exception that the workers demonstrated superior proficiency in recognizing temperature-related hazards—although this category of hazards is not highlighted as part of the Construction Focus Four program.

Third, although the workers demonstrated higher levels of proficiency in recognizing hazards in the gravity, motion, electrical, and temperature categories, on average, across these hazard categories, the workers only recognized roughly 47% of the safety hazards. In other words, despite the workers demonstrating relatively higher levels of proficiency in certain hazard categories, they still failed to recognize a substantial proportion of hazards even in the hazard categories where they demonstrated higher proficiency levels. In fact, the workers failed to recognize close to 40% of the safety hazards in the gravity hazard category where they demonstrated the highest proficiency levels. Given these findings, it is imperative that industry efforts such as the National Safety Stand-Down and the Construction Focus Four training program that largely target the hazards that workers are relatively more proficient in recognizing be maintained. In fact, this finding may suggest that these industry-level initiatives must be improved and better disseminated across the industry to achieve desirable hazard recognition levels. 

Finally, the findings of the current investigation reinforce previous findings that workers fail to recognize an unacceptable number of safety hazards [12,13,14]—whether in the category of higher proficiency or in the ones with lower proficiency levels. This finding suggests that new and improved hazard recognition interventions will be needed. Moreover, as per the current findings, as also discussed above, these interventions must target hazards in all categories—including the ones where workers demonstrated lower proficiency and the ones where relatively higher levels of proficiency were observed.

## 7. Study Limitations and Suggested Future Research

While the study presents findings related to the proficiency with which workers recognize hazards of different categories, there are some limitations that must be acknowledged. First, although the framework of energy sources adopted for the current investigation included a total of ten hazard categories (see Table 1), only seven of the hazard categories were included in the final investigation. This was because three of the hazard categories included a very small number of relevant safety hazards for making any useful statistical comparisons. Future efforts may seek to assess the proficiency that workers demonstrate in the context of these hazards that were under-represented in the hazard recognition task.

Second, the current investigation used construction case images that were captured from construction workplaces to administer the hazard recognition task. While such an approach provided a standardized and rigorous approach to assess proficiency without exposure to a real safety risk, future investigations in real work environments may provide additional advantages. For example, hazard recognition efforts that are conducted in real workplaces may better capture the dynamic and challenging nature of construction workplaces. Nonetheless, such an effort may introduce new challenges with offering a standardized approach to measure hazard recognition for workers in different workplaces and work environments. Moreover, apart from the expected safety risks and logistical challenges, additional procedures may be needed to assess performance—since a pre-identified list of recognized safety hazards, as was available for the current study with the support of a large panel of safety experts, may be unavailable. 

Third, while the current investigation provided evidence that workers are more proficient at recognizing certain categories of hazards than others, the research does not provide insights on why these differences occur. Although few probable explanations for these differences were discussed in the previous section (e.g., National Safety Stand-Down to Prevent Falls, Construction Focus Four training program), future empirical investigations that examine these particular hypotheses and others may be conducted. Such efforts may be beneficial given that little is known about the effectiveness of these industry-wide efforts for which significant investments are made every year.

Fourth, future research may focus on investigating some of the suggested improvements to existing hazard recognition interventions based on the current study findings. For example, future efforts may examine if highlighting the hazard categories that the workers were less proficient in recognizing—as an intervention in the JHA templates that workers use—leads to higher levels of attention towards these hazards. Likewise, the effect of highlighting these hazards as part of regular training efforts can be tested. The lessons learned from all these future efforts can all inform efforts to enhance hazard recognition levels in the construction industry.

Finally, while the current research offers evidence to suggest that workers are more proficient in recognizing certain hazards than others, future replications may be necessary to ensure that the findings are generalizable. Such replications will particularly be useful given that the current investigation only involved 287 workers compared to the large population of workers involved in the construction industry. Despite this limitation, it is important to note that the majority of the studies that have focused on construction hazard recognition, many of which have been referenced in the current article, involved fewer study participants than the currently reported study.

## 8. Conclusions

Effective hazard recognition is necessary for maintaining workplace safety. When workers fail to recognize and manage safety hazards, safety injuries, illnesses, and tragic incidents become more probable [12,14]. Unfortunately, evidence from the global construction industry suggests that workers fail to recognize a large proportion of safety hazards [12,13,14]. Such poor performance persists even when interventions such as job hazard analyses and safety training programs are adopted to facilitate hazard recognition [22,24].

Such failure to recognize workplace safety hazards can be partly attributed to important weaknesses in existing hazard recognition interventions. In fact, there is evidence to suggest that much of the interventions are developed with little understanding of the challenges workers experience during hazard recognition efforts [24]. Therefore, new and more robust efforts are needed to tackle the issue of poor hazard recognition in the construction industry.

Towards better understanding the challenges that workers experience during hazard recognition efforts, the current investigation focused on examining the hazard recognition patterns that workers demonstrate when participating in hazard recognition efforts. More specifically, the effort focused on examining if there were differences in the proficiency with which workers recognize hazards in different categories. The research objective was accomplished by recruiting 287 workers from 57 workplaces in the United States and engaging them in a hazard recognition activity.

The results of the study provided evidence that workers are more proficient in recognizing hazards in certain hazard categories over others. More specifically, the findings demonstrated that workers were more proficient in recognizing hazards in the gravity, electrical, motion, and temperature hazard categories over hazard in the pressure, chemical, and radiation hazard categories. 

The findings offer important suggestions to improve hazard recognition levels. For example, based on the findings, the industry may need to devote additional attention to enhancing hazard recognition in the hazard categories where workers demonstrated less proficiency (i.e., pressure, chemical, radiation). This can be possibly accomplished by offering more training that focuses on the less recognized hazards or by highlighting these hazards in the JHA templates that workers use. The findings also reveal that the attention devoted to hazards that workers are more proficient in recognizing (gravity, electrical, motion, and temperature) must also be maintained—since there is significant room for improvement.

## Figures and Tables

**Table 1:** Hazard categories based on the underlying energy sources and relevant examples.

Hazard Categories/Energy Sources	Example Hazards
Gravity	Object on the floor causing trip potential; slip on slippery surfaces, tools at height that can potentially fall; unprotected leading causing fall potential; work at height; etc.
Motion	Moving heavy equipment; dust carried by wind gusts; material transportation; etc.
Mechanical	Tools with rotating, moving, or vibrating components such a hand saw; conveyor belts; rotating shafts, drilling operations; etc.
Electrical	Overhead power lines; powered equipment and tools; unprotected electrical panels; etc.
Pressure	High-pressure cleaning equipment usage; compressed gas cylinders; unstable soil in a trench; etc.
Temperature	Steam; hot surfaces; cold surfaces; flammable substances; hot weather; etc.
Chemical	Construction material that contain carcinogens or toxic elements such as asbestos, lead-based paints, and other sources; chemical fumes; explosives, etc.
Biological	Insects; microorganisms, snakes, viruses, bacteria, spiders, mold, fungi, etc.
Radiation	Radiations from hot work, nuclear plants, and other sources; x-rays, low lighting, etc.
Sound	Equipment noise; pile driving operation; noise from blasting, etc.

**Table 2:** Demonstrated proficiency levels—descriptive and inferential statistics results.

Hazard Category	Mean	Std. Dev.	LLCI	ULCI	Welch’s Statistic	*p*-Value
Gravity	60.13%	27.26%	52.03%	68.22%	28.518	<0.05
Motion	42.62%	28.81%	31.22%	54.01%		
Electrical	44.49%	32.66%	19.38%	69.60%		
Pressure	15.21%	24.22%	14.87%	45.28%		
Temperature	42.73%	40.04%	14.09%	71.37%		
Chemical	8.54%	6.91%	4.36%	12.72%		
Radiation	5.46%	4.75%	−0.43%	11.36%		

Note: LLCI and ULCI = lower and upper limit confidence intervals.

**Table 3:** Pairwise comparisons of demonstrated proficiency levels across the hazard categories.

Pairwise Comparisons	Mean Difference	Standard Error	LLCI	ULCI
Gravity–Motion	17.51	6.835	−3.882	31.685
Gravity–Electrical	15.64	15.639	−7.046	39.789
Gravity–Pressure *	44.92	10.967	19.977	62.36
Gravity–Temperature	17.4	12.693	−7.147	41.999
Gravity–Chemical *	51.59	4.405	43.136	60.197
Gravity–Radiation *	54.67	4.498	45.929	63.936
Motion–Electrical	−1.87	12.165	−26.596	22.234
Motion–Pressure *	27.41	11.679	2.602	47.127
Motion–Temperature	−0.11	13.305	−25.493	25.794
Motion–Chemical *	34.08	5.927	21.903	45.661
Motion–Radiation *	37.16	5.883	25.563	48.874
Electrical–Pressure *	29.28	14.971	0.417	57.158
Electrical–Temperature	1.76	16.331	−28.488	34.676
Electrical–Chemical *	35.95	11.127	13.505	58.326
Electrical–Radiation *	39.03	11.257	17.2	60.988
Pressure–Temperature *	−27.52	16.1	−58.771	−5.961
Pressure–Chemical	6.67	10.469	−8.733	30.742
Pressure–Radiation	9.75	10.417	−5.832	32.119
Temperature–Chemical *	34.19	12.308	9.685	58.227
Temperature–Radiation *	37.27	12.286	13.256	61.026
Chemical–Radiation	3.08	2.952	−3.205	8.495

Note: LLCI and ULCI = lower and upper limit confidence intervals. * denotes that the pairwise comparison revealed a significant difference in proficiency levels.

**Table 4:** Summary results of pair-wise comparisons.

Hazard Categories	Groups	
	A	B
Gravity	x	
Electrical	x	
Motion	x	
Temperature	x	
Pressure		x
Chemical		x
Radiation		x
